# Comparing mortality in the elderly after proximal femur fractures and coxarthrosis: the effect of individual health characteristics and day of surgery

**DOI:** 10.1007/s00068-025-02882-y

**Published:** 2025-05-20

**Authors:** Anne Fink, Steffi S.I. Falk, Daniela Georges

**Affiliations:** 1 German Center for Neurodegenerative Diseases (DZNE), Bonn, Germany; 2 Department of Child and Adolescent Psychiatry, Rostock University Medical Center, Rostock, Germany; 3 Department of Trauma, Hand and Reconstructive Surgery, Rostock University Medical Center, Rostock, Germany; 4 Institute for Occupational, Social and Environmental Medicine, Rostock University Medical Center, Rostock, Germany; 5 Faculty of Economic and Social Sciences, Institute of Sociology and Demography, Chair of Empirical Methods in Social Science and Demography, University of Rostock, Rostock, Germany

**Keywords:** Surgery, Proximal femur fracture, Coxarthrosis, Geriatric, Mortality, Cox proportional models

## Abstract

**Purpose:**

This study investigates mortality variations between elective and urgent hip surgeries, focusing surgery timing and its impact on post-operative mortality. By comparing cases of femoral neck fractures, pertrochanteric fractures, and coxarthrosis across different follow-up durations, it aims to identify factors contributing to increased mortality.

**Methods:**

We used a random sample of German longitudinal health claims data (*N* = 250,000, 2004–2019) and identified 10,310 patients aged 50 years and older who underwent surgery for femoral neck fracture, pertrochanteric fracture, or coxarthrosis between 2004 and 2014. We tracked mortality at 30 days, 1 year, and 5 years. Cox proportional models were used, adjusted for the following covariates at the time of surgery: sex, age, comorbidities, nursing home dependency, discharge diagnosis, and weekday of surgery.

**Results:**

Mortality probabilities were 5% at 30 days, 15.6% at 1 year, and 38.9% at 5 years, with significantly higher risks for fractures than coxarthrosis. Key factors influencing mortality included age, comorbidities (e.g., heart failure, stroke, myocardial infarction, dementia), and care dependency levels. Women had lower risks than men across all periods. Short-term mortality was most affected by comorbidities, while long-term mortality correlated with chronic health conditions such as nicotine abuse and diabetes mellitus, and care needs. Surgery timing showed no consistent weekday effects.

**Conclusion:**

Mortality differences reflect the impact of acute trauma from emergency surgery rather than the surgical procedure itself, emphasizing the need for optimized planning, preparation, early treatment and adaptable care structures in an aging population.

**Supplementary Information:**

The online version contains supplementary material available at 10.1007/s00068-025-02882-y.

## Introduction

Hip fractures, particularly those of the femoral neck and pertrochanteric regions, constitute a significant and growing global health concern, characterized by considerable morbidity, mortality, and healthcare expenditure. These fractures predominantly affect geriatric and frail elderly, with low level falls and age-related bone mass reduction, typified by osteoporosis, being the most prevalent cause of hip fractures [1, 2]. Thus, as populations age, the incidence and prevalence of hip fractures have increased significantly and are projected to rise further, placing a substantial health burden on healthcare systems worldwide. The global incidence of hip fractures in individuals aged 55 and older reached 681.35 per 100,000 population in 2019, while the prevalence was recorded at 1,191.39 per 100,000 [1]. The rates (and absolute numbers) of hip fracture patients are particularly high and increasing in industrialized regions, such as Australasia, Western Europe, (high-income) North America and Central Europe [1]. In Germany, one of the oldest countries in the world, the number of femoral neck fractures (FNF) and pertrochanteric fractures (PTF) exceeded 150,000 in 2019, constituting the most common fractures [3].

Hip fractures are characterized by a high burden of disease, as they almost invariably require hospitalization and surgical treatment. Femoral fractures are typically classified based on their location and severity, which also dictate the choice of (surgical) treatment. Surgical strategies for FNF include internal fixation, hemiarthroplasty, and total hip arthroplasty. PTF, which are more prevalent in elderly patients, may be managed with either internal fixation, such as intramedullary nail or sliding/dynamic hip screw [4]. Surgical treatment of proximal femoral fractures includes osteosynthesis (dynamic screws, intramedullary nailing) and, in particular, the treatment of patients with a dual-mobility prosthesis and total hip joint endoprosthesis. The aim of all procedures is to achieve postoperative full weight-bearing for the patient’s affected hip. Consequently, coxarthrosis and proximal femur fractures frequently entail analogous surgical interventions, thus offering important potential for analysis, as the initial states and conditions differ considerably. While patients with a FNF or PTF typically experience an acute, traumatic event necessitating emergency surgery, patients with coxarthrosis generally undergo elective surgery, i.e. the operation and subsequent management can be planned.

Whilst the primary surgical interventions demonstrate efficacy, they are concomitant with adverse outcomes, including long-term loss of functional outcomes, subsequent care need, and excess mortality [5–10]. In-hospital mortality, primarily attributable to peri-operative complications such as respiratory or cardiac failure, ranges from 1.5 to 3.4% [11–13], and the 30-day mortality, when pre- and post-operative factors gain higher relevance, varies from 5.0 to 13.3% [11, 14]. The increase in mortality is particularly high within the first six months and thereafter decreases gradually [15]. Within the first year, approximately 20 to 30% of elderly hip fracture patients die [16–18]. Furthermore, although less extensively examined, there are (moderate) mortality disadvantages even beyond the first year [6, 8, 19]. Vestergaard et al. [20] demonstrate an excess mortality of 1.8% for every additional year following the 1-year period. However, peri-operative mortality and post-operative complications are significantly lower in patients undergoing coxarthrosis surgery compared to proximal femur fracture patients [15, 21, 22].

Post-operative outcomes substantially vary according to age and gender, pre-operative health conditions and comorbidities, cognitive function and accommodation (residential vs. community-dwelling) [5, 23–25]. Mortality rates were consistently and significantly higher in patients who were male, older, multimorbid, demented or cognitively impaired, and lived in nursing/residential care settings [26–28]. Furthermore, it is already well established that the time from admission to surgical intervention affects morbidity and mortality, with reduced mortality for patients operated within 24 h [29]. Consequently, according to the guidelines of the German Federal Joint Committee, treatment of patients with femoral fractures is required within 24 h [30]. Furthermore, early post-operative mobilization has been demonstrated to positively affect recovery and reduce mortality risks [31, 32]. In light of these findings, recent research has begun to explore how the day of the week may influence outcomes following hip fracture surgery. The so-called “weekend effect” has been demonstrated for many injuries and surgeries, with patients admitted during weekends experiencing worse clinical outcomes, including higher mortality rates [33–35]. Potential explanations for this pattern include extended waiting times for surgery, delayed interventions, diminished access to experienced and skilled surgical teams or physiotherapy, and a paucity of geriatric support. However, the findings on a weekend effect in hip surgery patients are inconclusive. Guo et al. [36], Daugaard et al. [37] and Nijland et al. [38] did not find significant differences in in-hospital, 30-day or long-term mortality or adverse outcomes for patients undergoing femoral fracture surgery on weekends or holidays compared to weekdays. However, Thomas et al. [39] reported a significant rise in 30-day mortality for patients admitted on weekends. In view of these conflicting findings, further research is required to determine the impact of surgical timing on post-hip fracture mortality.

Finally, a mortality and functional loss-reducing effect of early and appropriate geriatric rehabilitation has been reported [40–42], where particular importance is attached to adherence to rehabilitation programs and early mobilization [43]. Risk factors for non-adherence to early mobilization and early rehabilitation include poor pre- or post-surgery cognition, functional status/limitations, and disability and comorbidities [44–46].

The present study aims to further investigate the mortality differences observed in elective and urgent hip surgery patients and the potential impact of the day of admission on post-operative mortality. To expand the state of research in this area, we will contrast three groups of patients who underwent the same surgical procedures but for different initial diseases (FNF, PTF or coxarthrosis), and apply a short- (30 days), medium- (1 year), and long-term (5 years) follow-up period. This methodological approach provides significant insights into the magnitude, causes of and temporal pathways for excess mortality in hip fracture patients, which are highly relevant for informed clinical decision-making and improved patient outcomes.

## Methods

### Data

We used a random sample of longitudinal health claims data of persons aged 50 years or older from Germany’s largest public health insurance, the “Allgemeine Ortskrankenkasse” (AOK). The sample was drawn in the first quarter of the year 2004 (*n* = 250,000) and followed up until the end of 2019. The data contain information on sex, age, region of residence and, if applicable, date of death, as well as information on all reimbursed inpatient and outpatient diagnoses coded according to the German modification of the 10th revision of the International Statistical Classification of Diseases and Related Health Problems (ICD-10 [47]) All information was available on a quarterly basis.

### Ethics statement

This is an observational study which involved retrospective, anonymized claims data. It fell outside the scope of the Declaration of Helsinki and did not require ethical review.

Access to the data was legally approved by the Scientific Research Institute of the AOK (WIDO, granted on 4 February 2021). The study was based on administrative claims data in which patients were never directly involved and data were fully anonymized before analyses. Individual patients cannot be identified during or after data collection, and the analyses presented do not affect patients whose anonymized records were used. Participant consent was not required. The University of Rostock Research Ethics Committee confirmed that no ethical approval is required.

### Case selection: surgery with discharge diagnosis of fracture of femur neck, Pertrochanteric fracture, or coxarthrosis

To create our analysis sample, we first identified all patients with a discharge diagnosis of femoral neck fracture (ICD-10: S72.00, S72.01, S72.03-S72.05), pertrochanteric fracture (ICD-10: S72.1), or coxarthrosis (ICD-10: M16).

The inclusion criteria for the surgical cases were discussed with experts in the field of trauma, hand and reconstructive surgery (Rostock University Medical Center). Surgeries were defined according to the classification of surgical procedures [48]. The OPS codes used to identify surgical cases are shown in Table S1 (see the Supplementary Appendix).

We selected only patients with one of the above discharge diagnoses who underwent surgery between the beginning of 2004 and the end of 2014. This allowed us to have a minimum follow-up of 5 years. Furthermore, we only included patients with only one surgery during the observation period and those with less than 2 days between the day of hospital admission and the day of surgery according to the guidelines of the German Federal Joint Committee. We also excluded patients who were coded as having already had a hip replacement (ICD-10: Z96.64).

### Covariates

To adjust our models, we used following baseline covariates at time of the surgery: Sex (men, women), age in 5-year age groups (50–54, 55–59, 60–64, 65–69, 70–74, 75–79, 80–84, 85–89, 90+), neurodegenerative diseases such as dementia (ICD-10: F00-F03, G30, G310, G31.82, G23.1, F05.1, validated by applying an established validation strategy [49] and Parkinson’s disease (G20), mobility-impairing comorbidities after surgery such as heart failure (I09.9, I11.0, I13.0, I13.2, I25.5, I42.0, I42.5-I42.9, I43, I50, P29.0), stroke (I63, I64, I69.3, I69.4), myocardial infarction (MI; I21-I22, I25.2), and chronic obstructive pulmonary disease (COPD; J44), comorbidities or conditions, which may increase the risk for surgical site infections [50, 51], such as alcohol abuse (F10.1, F10.2), nicotine abuse (F17.1, F17.2), rheumatism (M05, M06, M31.5, M32-M34, M35.1, M35.3, M36.0), and diabetes mellitus (E10-E14); care need level defined as receiving benefits from the public German care insurance (eligibility is based on a standardized medical assessment by specialists who assigned care levels from 0 to 3 until 2016); nursing home placement; kind of discharge diagnosis (FNF, PTF, or coxarthrosis), and week day of the surgery.

### Definition of geriatric patients

The term ‘geriatric patient’ is defined in this paper as a patient over the age of 80. As the typical geriatric multimorbidity cannot be reliably determined from administrative data, the second criterion, age, was used for the definition [52].

### Definition of reference group

The reference patient used for the evaluation of the influencing factors analyzed was defined as follows: male, aged between 75 and 79 years, without comorbidities or care need dependency, not living in a nursing home, and undergoing surgery for a FNF on a Monday.

### Statistical analysis

We calculated the probability of dying after surgery by dividing the number of deaths within a predefined observation period (5 years, 1 year, or 30 days) by the total number of patients who underwent first surgery for FNF, PTF, or coxarthrosis between 2004 and 2014.

Cox proportional hazards models were used to examine the risk of death after hip surgery within 5 years, 1 year, and 30 days. Time was measured in days from the date of surgery. Patients were followed until the date of death, attrition (due to change of public health insurance), or end of observation (after 5 years, 1 year, or 30 days), whichever occurred first.

As sensitivity analyses, we stratified the full models by age to distinguish between non-geriatric patients (age < 80 years) and geriatric patients (age > = 80 years). We also looked only at the discharge diagnoses of FNF and PTF and excluded all patients who underwent surgery for coxarthrosis.

All analyses were performed using Stata MP 16.1 (StataCorp LLC).

## Results

Table 1 displays the number of included patients (*N* = 10,310) aged 50 years and above at time of the surgery, as well as the mortality probabilities in percent within 5 years, 1 year, and 30 days after surgery, along with their respective lower and upper confidence intervals (LCI and UCI). Overall, we observed a probability of death of 38.9% (95% confidence interval [58.5–60.4%]) within 5 years, 15.6% [14.9-16-3%] within 1 year, and 5% [4.6–5.4%] within 30 days, with fundamental differences between surgery for fractures and surgery for coxarthrosis. Patients who underwent surgery for fractures had a 22-fold higher probability of death within 30 days, an 18-fold higher probability of death within 1 year, and almost a 6-fold higher probability of death within 5 years. Men and women did not differ significantly in their 1-year and 30-day probability of death, but at 5 years, women had slightly higher probabilities than men. As expected, the probability of death for all three observation periods increased significantly with age at the time of surgery. The presence of all included potentially mobility-impairing comorbidities (dementia, Parkinson’s disease, heart failure, stroke, MI, and COPD) significantly increased the probability of death, with the effects most pronounced when looking at 30-day mortality. The presence of alcohol abuse, nicotine abuse, and rheumatism were not associated with a higher probability of death. However, the presence of diabetes mellitus led to significantly increased death probabilities for all three observation periods. The higher the level of care dependency at the time of surgery, the higher the probability of death within 1 and 5 years. With regard to 30-day mortality, only patients without long-term care dependency had a significantly lower probability of death. Residence in a nursing home significantly increased the probability of death: 82.7% [80.5–84.7%] of all nursing home residents had died at 5 years, compared with 32.6% [31.7–33.6%] for non-residents. The day of the week on which surgery was performed showed increased probabilities for the weekend, which can be attributed to the reason for surgery, with surgery for coxarthrosis rarely performed on the weekend.

**Table 1:** Description of study population and probability of mortality 5 years, 1 year and 30 days after hip surgery with 95% confidence interval. Source: AOK data 2004–2019.

				5-year mortality			1-year mortality			30-day mortality		
Variable		*N*	%	Probability in %	LCI	UCI	Probability in %	LCI	UCI	Probability in %	LCI	UCI
Sex	Men	3,126	30.3	55.92	54.17	57.65	15.58	14.35	16.89	5.34	4.61	6.19
	Women	7,184	69.7	61.00	59.86	62.12	15.63	14.81	16.49	4.82	4.34	5.34
Age group	50–54	96	0.9	21.88	14.72	31.24	1.04	0.15	7.02	1.04	0.15	7.02
	55–59	393	3.8	19.85	16.20	24.09	2.80	1.56	4.98	0.25	0.04	1.78
	60–64	735	7.1	23.27	20.35	26.46	2.86	1.87	4.34	0.41	0.13	1.26
	65–69	1,145	11.1	30.13	27.54	32.85	3.84	2.87	5.13	0.52	0.24	1.16
	70–74	1,755	17.0	40.80	38.52	43.12	5.53	4.55	6.70	1.48	1.01	2.17
	75–79	1,974	19.2	57.70	55.51	59.86	9.47	8.26	10.85	3.04	2.37	3.90
	80–84	1,805	17.5	78.34	76.38	80.18	18.28	16.57	20.13	5.10	4.17	6.21
	85–89	1,324	12.8	90.33	88.62	91.81	30.74	28.31	33.28	9.82	8.33	11.54
	90+	1,083	10.5	96.95	95.74	97.83	47.28	44.32	50.26	17.91	15.74	20.31
Dementia	No	7,807	75.7	49.16	48.05	50.27	9.00	8.39	9.66	2.88	2.53	3.28
	Yes	2,503	24.3	91.57	90.42	92.60	36.24	34.38	38.14	11.51	10.31	12.82
Parkinson’s disease	No	9,813	95.2	58.14	57.16	59.11	15.02	14.33	15.74	4.76	4.35	5.20
	Yes	497	4.8	85.51	82.14	88.34	27.36	23.62	31.45	9.26	7.00	12.14
Heart failure	No	6,243	60.6	48.60	47.36	49.84	8.63	7.96	9.36	2.21	1.87	2.61
	Yes	4,067	39.5	76.12	74.79	77.41	26.33	25.00	27.71	9.22	8.37	10.15
Stroke and/or MI	No	8,372	81.2	55.33	54.26	56.39	12.98	12.28	13.72	3.94	3.55	4.38
	Yes	1,938	18.8	77.30	75.38	79.11	26.99	25.06	29.01	9.44	8.22	10.83
COPD	No	8,457	82.0	57.89	56.84	58.94	14.37	13.64	15.13	4.48	4.06	4.94
	Yes	1,853	18.0	66.59	64.41	68.71	21.32	19.51	23.24	7.23	6.14	8.50
Alcohol abuse	No	10,085	97.8	59.25	58.28	60.20	15.59	14.89	16.31	5.02	4.61	5.46
	Yes	225	2.2	68.89	62.54	74.60	16.89	12.54	22.36	3.11	1.49	6.38
Nicotine abuse	No	9,998	97.0	59.74	58.76	60.72	15.64	14.92	16.38	5.00	4.58	5.45
	Yes	312	3.0	55.47	51.73	59.16	15.33	12.82	18.23	4.67	3.32	6.53
Rheumatism	No	9,484	92.0	59.46	58.47	60.44	15.75	15.03	16.50	5.06	4.64	5.52
	Yes	826	8.0	59.44	56.06	62.74	14.04	11.84	16.58	4.00	2.85	5.57
Diabetes mellitus	No	6,574	63.8	54.87	53.66	56.07	13.29	12.50	14.14	4.08	3.62	4.58
	Yes	3,736	36.2	67.53	66.01	69.02	19.70	18.46	21.01	6.56	5.81	7.40
Care need level	0	6,962	67.5	43.88	42.72	45.05	5.47	4.96	6.03	1.69	1.42	2.03
	1	1,563	15.2	87.72	85.99	89.25	29.37	27.16	31.67	11.00	9.55	12.65
	2	1,439	14.0	94.72	93.44	95.76	41.07	38.55	43.63	13.00	11.35	14.83
	3	346	3.4	98.55	96.58	99.40	51.73	46.47	56.96	10.40	7.60	14.09
Nursing home	No	9,027	87.6	54.26	53.23	55.29	12.34	11.68	13.04	4.10	3.71	4.53
	Yes	1,283	12.4	96.02	94.81	96.97	38.66	36.03	41.36	11.15	9.54	12.99
Discharge diagnosis	S72.0	2,153	20.9	82.30	80.63	83.86	27.59	25.74	29.52	8.82	7.70	10.10
	S72.1	2,941	28.5	86.91	85.64	88.08	31.38	29.73	33.09	10.20	9.16	11.35
	M16	5,216	50.6	34.55	33.27	35.85	1.78	1.46	2.18	0.44	0.29	0.66
Date of week, surgery	Sunday	619	6.0	83.68	80.56	86.39	28.11	24.71	31.78	8.56	6.60	11.04
	Monday	1,569	15.2	55.51	53.04	57.96	14.21	12.57	16.03	4.59	3.66	5.74
	Tuesday	2,012	19.5	54.87	52.69	57.03	13.12	11.71	14.67	4.37	3.56	5.36
	Wednesday	1,972	19.1	54.26	52.05	56.45	13.29	11.86	14.86	4.61	3.77	5.63
	Thursday	1,862	18.1	54.67	52.40	56.92	12.08	10.68	13.64	3.87	3.08	4.84
	Friday	1,650	16.0	62.00	59.63	64.31	17.15	15.41	19.05	4.85	3.91	6.00
	Saturday	626	6.1	84.03	80.94	86.69	28.59	25.19	32.26	9.11	7.09	11.62
**Total**		**10**,**310**	**100.0**	**59.46**	**58.51**	**60.40**	**15.62**	**14.93**	**16.33**	**4.98**	**4.57**	**5.41**

UCI: upper confidence interval, LCI: lower confidence interval, MI: myocardial infection, COPD: chronic obstructive pulmonary disease, S72.0: femoral neck fracture, S72.1: pertrochanteric fracture, M16: coxarthrosis

### Model results

Table 2 shows the results of the full Cox model for the risk of mortality in terms of hazard ratios (HR) at 5 years, 1 year, and 30 days after surgery. Adjusted for covariates, we found that women had a significantly lower risk of death compared with men for all three observation periods (5 years: HR = 0.57 [0.53–0.62]; 1 year: HR = 0.54 [0.48–0.61]; 30 days: HR = 0.52 [0.42–0.63]). As expected, we observed an age gradient with an increasing risk of death with increasing age at the time of surgery, e.g. with regard to 5-year mortality, patients aged 65 to 69 years had a 50% lower risk of death compared to patients aged 75 to 79 years (HR = 0.50 [0.42–0.60]), whereas patients aged 85 to 89 years had a 60% increased risk of death compared to patients aged 75 to 79 years (HR = 1.60 [1.43–1.78]). Adjusted for demographics, comorbidities, and functional status, the presence of dementia was associated with an increased risk of death at 1 year (HR = 1.17 [1.04–1.32]) and 5 years (HR = 1.25 [1.15–1.35]), but not in the short term of 30 days. The presence of Parkinson’s disease was not associated with mortality regardless of the length of follow-up. All included potentially mobility-impairing comorbidities such as heart failure, stroke and/or myocardial infarction, and COPD significantly increased the risk of death in all three observation periods, with the effects being most pronounced in the short term of 30 days (heart failure: HR = 1.81 [1.47–2.22]; stroke and/or MI: HR = 1.26 [1.04–1.52]; COPD: HR = 1.27 [1.03–1.56]. Among the conditions that may increase the risk of infection, we found no significant effects in the short and medium term, but in the long term of 5 years with increased risks for nicotine abuse (HR = 1.32 [1.09–1.61]), and diabetes mellitus (HR = 1.10 [1.03–1.18]). Higher care need levels were strongly correlated with increased mortality risks at 5 years and 1 year, e.g. care need level 3 at 5 years: HR = 2.74 [2.37–3.17]. In the short term, care need levels 1 and 2 were associated with increased risks (level 1: HR = 1.69 [1.30–2.20]; level 2: HR = 1.67 [1.27–2.21]), whereas there was no significant effect for care need level 3. Nursing home placement was not associated with an increased risk of mortality at any of the time intervals examined. Across all time intervals examined, surgery for coxarthrosis (M16) was associated with a significantly reduced risk of death compared to surgery for FNF (S72.0) or PTF (S72.1) (e.g. 30 days: HR = 0.13 [0.08–0.22]). The evaluation of the day of the week showed no significant differences in the short or medium term. Only at 5 years, we found a significant effect with patients who underwent surgery on Saturday had a significantly reduced risk of mortality compared to patients who underwent surgery on Monday (HR = 0.87 [0.77–0.99]).

**Table 2:** Results of Cox regression models. Risk of mortality in terms of hazard ratios (HR) 5 years, 1 year and 30 days after hip surgery with 95% confidence interval. Source: AOK data 2004–2019.

		5-year mortality					1-year mortality					30-day mortality			
Variable		HR	*p*	LCI	UCI		HR	*p*	LCI	UCI		HR	*p*	LCI	UCI
Sex	Women (Ref. Men)	0.64	< 0.001	0.60	0.68		0.54	< 0.001	0.48	0.61		0.52	< 0.001	0.42	0.63
Age group	50–54	0.29	< 0.001	0.19	0.46		0.15	0.057	0.02	1.05		0.54	0.542	0.07	3.99
	55–59	0.32	< 0.001	0.25	0.41		0.57	0.076	0.31	1.06		0.18	0.091	0.02	1.31
	60–64	0.39	< 0.001	0.33	0.45		0.51	0.004	0.32	0.80		0.24	0.017	0.07	0.77
	65–69	0.52	< 0.001	0.46	0.59		0.66	0.017	0.47	0.93		0.30	0.005	0.13	0.70
	70–74	0.71	< 0.001	0.64	0.78		0.82	0.112	0.64	1.05		0.69	0.121	0.43	1.10
	75–79 (Ref.)	1.00					1.00					1.00			
	80–84	1.23	< 0.001	1.14	1.34		1.21	0.038	1.01	1.46		1.02	0.921	0.73	1.42
	85–89	1.57	< 0.001	1.44	1.72		1.54	< 0.001	1.29	1.85		1.52	0.010	1.10	2.08
	90+	2.11	< 0.001	1.92	2.32		2.30	< 0.001	1.92	2.75		2.56	< 0.001	1.88	3.50
Dementia	Yes (Ref. No)	1.20	< 0.001	1.12	1.29		1.17	0.008	1.04	1.32		1.17	0.128	0.96	1.42
Parkinson’s disease	Yes (Ref. No)	1.01	0.826	0.91	1.12		0.92	0.385	0.77	1.11		1.09	0.588	0.80	1.49
Heart failure	Yes (Ref. No)	1.21	< 0.001	1.15	1.28		1.53	< 0.001	1.37	1.71		1.81	< 0.001	1.47	2.22
Stroke and/or MI	Yes (Ref. No)	1.18	< 0.001	1.11	1.25		1.15	0.009	1.04	1.29		1.26	0.016	1.04	1.52
COPD	Yes (Ref. No)	1.17	< 0.001	1.09	1.24		1.28	< 0.001	1.13	1.44		1.27	0.022	1.03	1.56
Alcohol abuse	Yes (Ref. No)	1.26	0.007	1.07	1.49		1.10	0.582	0.78	1.57		0.86	0.709	0.39	1.91
Nicotine abuse	Yes (Ref. No)	1.30	0.001	1.11	1.52		1.22	0.205	0.90	1.67		1.24	0.464	0.69	2.23
Rheumatism	Yes (Ref. No)	0.97	0.518	0.88	1.06		0.93	0.482	0.77	1.13		0.80	0.223	0.56	1.14
Diabetes mellitus	Yes (Ref. No)	1.08	0.005	1.02	1.14		1.09	0.103	0.98	1.20		1.17	0.080	0.98	1.40
Care need level	0 (Ref.)	1.00					1.00					1.00			
	1	1.48	< 0.001	1.36	1.60		1.73	< 0.001	1.48	2.02		1.69	< 0.001	1.30	2.20
	2	1.84	< 0.001	1.68	2.01		2.14	< 0.001	1.82	2.52		1.67	< 0.001	1.27	2.21
	3	2.48	< 0.001	2.17	2.84		2.83	< 0.001	2.29	3.48		1.27	0.257	0.84	1.91
Nursing home	Yes (Ref. No)	1.04	0.308	0.96	1.12		0.93	0.229	0.83	1.05		0.81	0.058	0.66	1.01
Discharge diagnosis	S72.0 (Ref.)	1.00					1.00					1.00			
	S72.1	1.03	0.353	0.97	1.09		1.06	0.286	0.95	1.17		1.04	0.645	0.87	1.25
	M16	0.46	< 0.001	0.42	0.50		0.14	< 0.001	0.11	0.18		0.13	< 0.001	0.08	0.22
Date of week, surgery	Sunday	0.88	0.024	0.79	0.98		0.84	0.089	0.69	1.03		0.82	0.286	0.58	1.18
	Monday (Ref.)	1.00					1.00					1.00			
	Tuesday	1.03	0.566	0.94	1.12		1.01	0.885	0.85	1.21		1.06	0.707	0.78	1.45
	Wednesday	0.97	0.520	0.89	1.06		0.97	0.751	0.81	1.16		1.09	0.567	0.80	1.49
	Thursday	0.93	0.138	0.85	1.02		0.91	0.293	0.75	1.09		0.95	0.762	0.69	1.32
	Friday	1.00	0.916	0.92	1.10		1.01	0.951	0.84	1.20		0.90	0.505	0.65	1.23
	Saturday	0.90	0.062	0.81	1.01		0.89	0.263	0.73	1.09		0.91	0.576	0.64	1.28
Number of patients		10,310					10,310					10,310			
Number of deaths		6130					1610					513			

HR: Hazard ratio, UCI: upper confidence interval, LCI: lower confidence interval, Ref.: Reference group, MI: myocardial infection, COPD: chronic obstructive pulmonary disease, S72.0: femoral neck fracture, S72.1: pertrochanteric fracture, M16: coxarthrosis

Sensitivity analyses revealed similar results and trends for subgroups (Tables S2-S4). In the most vulnerable and affected group of geriatric patients aged 80 years and older, the presence of heart failure was significantly associated with increased 30-day mortality (HR = 1.77 [1.40–2.23]). At 1 year, the presence of dementia (HR = 1.20 [1.06–1.37]), heart failure (HR = 1.43 [1.26–1.62]), stroke and/or myocardial infarction (HR = 1.17 [1.04–1.33]), and COPD (HR = 1.20 [1.04–1.38]) significantly increased the risk of death (Table S3).

## Discussion

This study presents a 30-day, 1-year, and 5-year mortality follow-up comparing patients who underwent surgical intervention after femoral neck fracture (FNF), pertrochanteric fracture (PTF), and coxarthrosis. The results illustrate considerable post-surgical mortality risks even beyond the usually reported 12-month period. However, these risks vary significantly across patient groups, follow-up period, and pre-operative health conditions. In contrast, no clear effect of the week day of admission could be identified.

The probability of death was 5% within 30 days, 15.6% within 1 year, and 38.9% within 5 years. The 30-day and 1-year figures essentially align with the findings from other studies [11, 14, 16–18], although they fall at the lower limit of the reported probability range. This discrepancy may be attributable to several methodological and population-related factors. Firstly, our analysis is based on health claims data, which provide a comprehensive coverage of the patient population less influenced by selection bias. Secondly, the utilization of more recent data enables the consideration of potential advancements in peri-operative care and post-operative rehabilitation, which may not have been fully considered in earlier studies. Other studies have also reported declining or decreased mortality rates in hip surgery patients: Hao et al. [53] reported a 1-year mortality risk of 8.7%, [54] of 16.6%, and Downey et al. [16] of 22%, with a decrease over time. Thirdly, the present study incorporated coxarthrosis and PTF patients, who have been documented to exhibit comparably low risks of post-surgical complications [15, 21, 22, 55]. Fourthly, our analysis encompassed a young sample (patients aged 50 years and above). Our and earlier findings [55] demonstrate significantly lower mortality rates in younger individuals. Fifth, we only included patients who underwent surgery within one day of admission, which excludes patients whose surgery had to be postponed due to a complication, which in turn may result in higher mortality. Finally, considering the regional variability in post-operative mortality following hip surgery [56] —which reflects differences in medical care— cross-country comparison is only partially justifiable. Moreover, the present study is among a limited number of research endeavors that offer insights into 5-year mortality. The observed mortality rate of 38.9% is not exclusively attributable to hip fracture or hip surgery, but both the general figure and the age-specific figures exceeded the general death probability in these age group(s) [57].

Most importantly, our findings indicate that the surgical intervention itself is not the primary factor contributing to mortality in patients with hip fracture or coxarthrosis. Rather, the acute nature of the event/accident and the urgency in the procedure appear to be more relevant factors. Patients with coxarthrosis, who typically undergo elective and predictable surgical interventions, exhibited significantly lower mortality probabilities and risks, aligning with prior findings [15, 21, 22]. These outcomes were evident across all three follow-up periods examined, with the greatest reductions observed in the 30-day (by 87% compared to FNF patients) and 1-year period (by 86%). These results underscore the pivotal role of surgical urgency in determining post-surgical outcomes, influenced by a multifaceted array of factors. These include the selection of an opportune and optimal time for the patient and the medical team, meticulous pre-operative preparation, and comprehensive post-operative management and aftercare. The presence of these characteristics is often associated with reduced rates of surgical complications [30]. A comparative analysis of patients with FNF and PTF reveals no significant disparities in terms of mortality risk, thereby substantiating these assumptions.

The day of the week had a varied impact. The crude probabilities of death were found to be significantly elevated following surgical procedures conducted on Saturday or Sunday. However, when adjusting for relevant confounders, these effects became largely insignificant. This can be attributed to the fact that, in contrast to elective coxarthroses, acute cases are predominantly treated during the weekend. In the multivariate analysis conducted, the impact of the week day of the surgery on short-term mortality (30 days) was found to be negligible across all models. However, in the 1-year period, interventions conducted on Sundays were associated with a borderline significant mortality benefit (*p* = 0.089; HR = 0.84 [0.69–1.04]). Further analyses revealed that this effect was attributable exclusively to patients with FNF and PTF (HR = 0.82 [0.67-1.00]). In the long term (5 years), surgeries conducted on Saturdays resulted in reduced mortality risks [HR = 0.87 [0.77–0.99]), a finding that was only evident in the group of patients below the age of 80 (HR = 0.76 [0.59-1.00]) and in those patients with FNF and PTF (HR = 0.84 [0.73–0.96)]. Furthermore, the latter group demonstrated a reduced mortality risk when undergoing surgery on Sunday (HR = 0.86 [0.76–0.99]) or Thursday (HR = 0.85 [0.75–0.97]). These inconclusive results suggest that there is no clear weekday effect and that the weekend effect, if existent, is non-short-term and potentially even inverse. This finding aligns with the hypothesis that post-operative outcomes are influenced by factors beyond the weekday of surgery [37, 38, 58]. However, it is important to note that the study’s sample consists exclusively of patients with less than two days between the day of hospital admission and the day of surgery. Consequently, these findings underscore the significant mitigating effect of immediate surgery on the potential impact of weekend submission. The findings underscore the efficacy of expeditious remobilization and geriatric follow-up care, irrespective of the day of surgery [40–43].

Pre-operative health status was identified as a substantial predictor of mortality, aligning with prior research [5, 23]. The presence of heart failure was associated with an elevated mortality risk ranging from 77 to 85% within the first 30 days. However, this effect diminished over time, reaching 23 to 42% after 5 years, indicating a healthy survivor bias. In contrast, the mortality-increasing effects of COPD and stroke and/or MI remained relatively constant over time, ranging from 15 to 28% in the full sample. Nonetheless, these effects exhibited notable variations according to age. While COPD was predominantly relevant in the younger age group (< 80 years) and increased its 30-day mortality risk particularly strong (HR = 1.87 [1.21–2.87]), stroke and/or MI affected mortality disadvantages by 17–25% in those aged 80 years and older. Dementia was associated with an increased mortality risk by 17–25%, but only in the medium term and long term. Parkinson’s disease, on the other hand, demonstrated no significant impact on mortality risks, which may be attributed to the high intercorrelation of Parkinson’s disease and the other comorbidities included. The analysis further revealed that comorbidities or conditions, which may increase the risk for surgical site infections, could hardly be identified as defining 30-day or 1-year mortality risks in hip surgery patients, with the exception of diabetes in the group aged < 80 years. In contrast, nicotine abuse, diabetes mellitus and alcohol abuse were associated with increased 5-year mortality risk. The impact of alcohol abuse exhibited notable variations across the two age groups, demonstrating a 45% increased 5-year mortality risk among individuals under 80 years and a 38% decreased risk among those over 80 years (*p* = 0.085). Rheumatism did not predict mortality at all. These observations underscore the minimal influence of infection-promoting comorbidities on short-term and medium-term outcomes, and suggest delayed manifestation of behavioral health characteristics. The findings underscore the significance of physical health and physical activity in recovery processes. Furthermore, they highlight the necessity for extended follow-up periods in studies assessing health outcomes in hip injury patients and the importance of customized pre-, peri- and post-operative risk assessments that extend beyond conventional comorbidity indices.

The degree of prevalent care need dependency was associated with mortality differences, however, living in a nursing home was largely not associated with these differences when controlling for care need dependency. With regard to the care need level, a clear positive association was observed within 1 year and 5 years. However, within the first 30 days, the results were less clear, as the highest care level was not significantly associated with higher mortality risk. Living in a nursing home was associated with a short-term mortality reduction (*p* = 0.058). Despite contradicting prior research [26, 27, 59] and findings of the negative impact of frailty and multimorbidity on mortality outcomes [27, 28], this could indicate that either patients with severe care need or admitted from nursing homes who underwent surgical fracture treatment were (health) selected or that these individuals receive faster and better geriatric follow-up care in the short term after hospital discharge. Moreover, our models were adjusted for several comorbidities and nursing home status, which are correlated with (the assessment of) care need and care level in Germany [60].

Finally, the findings confirm gender- and age-specific disparities in mortality outcomes following hip surgery [26–28]. The mortality risk exhibited an increasing trend with advancing age, and the (adjusted) mortality risk among female patients was found to be nearly half that of their male counterparts. The comparison between the crude probability of death and adjusted mortality risks clearly demonstrates that women have a disadvantageous health profile and age structure that contributes significantly to mortality differences.

### Limitations

This study offers significant insights into mortality outcomes following hip fracture; however, several limitations must be considered when interpreting the results.

Firstly, the analysis focused on effect of the week day; however, the timing of surgery, such as off-hour admissions, was not analyzed due to missing information in the data. Some studies have suggested an association between off-hour surgery and mortality risk; however, findings specific to hip fracture surgery remain inconclusive. For instance, Zhou et al. [61] and Switzer et al. [62] reported no association. Forssten et al. [63] observed increased 30- and 90-day mortality following out-of-hours surgical interventions, but only in patients undergoing arthroplasty, not in those who received internal fixation.

Secondly, limitations pertain to the measurement of comorbidities and health status. This study focused on specific diagnoses and conditions at baseline. Consequently, the impact of subsequent diseases that emerge after the quarter of the surgery, which may also influence the mortality risk in the medium and long term, was not captured. Prior studies have employed the Charlson Comorbidity Index to predict mortality in hip surgery patients [62]. Sensitivity analyses yielded similar results (not shown) regarding the effect of sex, age and day of the week when using the index. However, these analyses were less informative because we do not know which comorbidity leads to an increase in the index. Consequently, we chose to use selected diseases, as this information may be more pertinent to clinical practice. Additionally, the ASA (American Society of Anesthesiologists) score was not employed, as it assesses surgical risks [64] but not long-term (mortality) risks. Moreover, subjective indicators, including but not limited to physical, psychological, or cognitive impairments, and socio-demographic characteristics, such as education or ethnicity [27], were excluded due to their absence in health claims data. In comparison with other data sources, health claims data provided an exceptional and unbiased foundation for our analyses, as nearly all patients with hip fractures require hospitalization and are thus captured in health claims data.

Thirdly, the data did not permit the complete assessment of post-operative complications, particularly prosthetic infections, beyond the measurement of comorbidity. Although infection-promoting factors (alcohol abuse, nicotine abuse, rheumatism, and diabetes) were controlled for, infections themselves were not explicitly analyzed. Post-operative infections are complex and influenced by numerous factors such as surgical duration, surgical team, peri-operative antibiotic prophylaxis timing, and soft tissue damage severity [50, 51]. Given the unavailability of these parameters in the data, the study may not fully capture the impact of infections on long-term mortality outcomes.

Notwithstanding these limitations, the study provides a robust analysis of mortality risks following hip fracture of femur neck, pertrochanteric fracture, or coxarthrosis, leveraging large-scale health claims data that encompass nearly all hospitalized cases. Future research should incorporate more detailed clinical parameters.

## Conclusions

The significant variations in mortality observed between femoral neck fracture or pertrochanteric fracture and coxarthrosis imply that the effect on health outcomes is not attributable to surgical intervention for hip fractures, but rather to the acute trauma associated with emergency surgery. This finding underscores the critical importance of meticulous planning and preparation for surgery and post-operative care. In the context of demographic ageing, accompanied by an increase in age-related diseases such as femoral neck fractures, healthcare facilities and post-operative care structures must adapt to enhance patient care. While pre-operative health conditions are significantly linked to post-operative outcomes, the day of the week of surgery is not. This finding underscores the significance of early surgical treatment, superseding the timing of the intervention.

## Electronic supplementary material

Below is the link to the electronic supplementary material.

Supplementary Material 1

Supplementary Material 2

Supplementary Material 3

Supplementary Material 4

## Data Availability

The Scientific Research Institute of the AOK (WIdO) imposes strict rules on sharing health claims data, as these are classified according to ethical restrictions due to privacy concerns. Anonymized data are available to researchers and institutions upon request. In order to request access to the health claims data of the AOK, please contact the WIdO directly (http://www.wido.de/, mail: wido@wido.bv.aok.de).
