# Supplementary material for: Comparing mortality in the elderly after proximal femur fractures and coxarthrosis: the effect of individual health characteristics and day of surgery
Source: Eur J Trauma Emerg Surg. 2025 May 20;51(1):213. doi: 10.1007/s00068-025-02882-y (PMC12092511; doi:10.1007/s00068-025-02882-y)
Supplement: Supplementary file 1 — Supplementary Material 1 [file 68_2025_2882_MOESM1_ESM.docx]

Table S1: OPS codes used to identify surgical cases

| OPS-Code & Description | |
| --- | --- |
| 5-820 Implantation of an endoprosthesis in the hip joint | |
| 5-820.0 | Total endoprosthesis |
| 5-820.2 | Total endoprosthesis, custom prosthesis |
| 5-820.4 | Dual-mobility prosthesis |
| 5-820.5 | Joint socket support shell |
| 5-820.7 | Joint snap cup |
| 5-790 Closed repositioning of a fracture with osteosynthesis | |
| 5-790.0 e/f | By screw Neck of femur/Proximal Femur |
| 5-790.3 e/f | By intramedullary nail with drilling of the medullary cavity Neck of femur/Proximal Femur |
| 5-790.4 e/f | By inter-locking nail Neck of femur/Proximal Femur |
| 5-790.5 e/f | By intramedullary nail with joint component Neck of femur/Proximal Femur |
| 5-790.7 e/f | By fixed angle plate/condylar plate Neck of femur/Proximal Femur |
| 5-790.8 e/f | By dynamic compression screw Neck of femur/Proximal Femur |
| 5-790.k e/f | By angular stable plate Neck of femur/Proximal Femur |
| 5-793 Repositioning of fracture: Open repositioning of a simple fracture in the joint area of a long bone | |
| 5-793.1 e/f | By screw Neck of femur/Proximal Femur |
| 5-793.2 e/f | By wire or tension band wiring/cerclage Neck of femur/Proximal Femur |
| 5-793.3 e/f | By plate Neck of femur/Proximal Femur |
| 5-793.4 e/f | By fixed angle plate/condylar plate Neck of femur/Proximal Femur |
| 5-793.5 e/f | By dynamic compression screw Neck of femur/Proximal Femur |
| 5-793.a e/f | By intramedullary nail with joint component Neck of femur/Proximal Femur |
| 5-794 Repositioning of fracture: Open repositioning of a multiple fragment fracture in the joint area of a long bone | |
| 5-794.0 e/f | By screw Neck of femur/Proximal Femur |
| 5-794.1 e/f | By wire or tension band wiring/cerclage Neck of femur/Proximal Femur |
| 5-794.2 e/f | By plate Neck of femur/Proximal Femur |
| 5-794.3 e/f | By fixed angle plate/condylar plate Neck of femur/Proximal Femur |
| 5-794.4 e/f | By dynamic compression screw Neck of femur/Proximal Femur |
| 5-794.a e/f | By intramedullary nail with joint component Neck of femur/Proximal Femur |
| 5-794.k e/f | By angular stable plate Neck of femur/Proximal Femur |
| 5-786.6 By intramedullary nail with joint component | |
