# Supplementary material for: Comparing mortality in the elderly after proximal femur fractures and coxarthrosis: the effect of individual health characteristics and day of surgery
Source: Eur J Trauma Emerg Surg. 2025 May 20;51(1):213. doi: 10.1007/s00068-025-02882-y (PMC12092511; doi:10.1007/s00068-025-02882-y)
Supplement: Supplementary file 2 — Supplementary Material 2 [file 68_2025_2882_MOESM2_ESM.docx]

Table S2: Results of Cox regression models for patients aged 50 to 79 years. Risk of mortality in terms of hazard ratios (HR) 5 years, 1 year and 30 days after hip surgery with 95 % confidence interval. Source: AOK data 2004-2019.

|  |  | **5-year mortality** | | | |  | **1-year mortality** | | | |  | **30-day mortality** | | | |
| --- | --- | --- | --- | --- | --- | --- | --- | --- | --- | --- | --- | --- | --- | --- | --- |
| **Variable** |  | **HR** | **p** | **LCI** | **UCI** |  | **HR** | **p** | **LCI** | **UCI** |  | **HR** | **p** | **LCI** | **UCI** |
| Sex | Women (Ref. Men) | 0.66 | <0.001 | 0.60 | 0.71 |  | 0.53 | <0.001 | 0.43 | 0.66 |  | 0.51 | 0.002 | 0.33 | 0.79 |
|  |  |  |  |  |  |  |  |  |  |  |  |  |  |  |  |
| Age group | 50-54 | 0.30 | <0.001 | 0.19 | 0.46 |  | 0.15 | 0.064 | 0.02 | 1.12 |  | 0.66 | 0.685 | 0.09 | 5.03 |
|  | 55-59 | 0.33 | <0.001 | 0.26 | 0.41 |  | 0.59 | 0.105 | 0.32 | 1.12 |  | 0.20 | 0.118 | 0.03 | 1.50 |
|  | 60-64 | 0.39 | <0.001 | 0.33 | 0.46 |  | 0.52 | 0.007 | 0.32 | 0.84 |  | 0.24 | 0.020 | 0.07 | 0.80 |
|  | 65-69 | 0.53 | <0.001 | 0.46 | 0.60 |  | 0.67 | 0.025 | 0.47 | 0.95 |  | 0.30 | 0.007 | 0.13 | 0.72 |
|  | 70-74 | 0.71 | <0.001 | 0.64 | 0.78 |  | 0.84 | 0.169 | 0.65 | 1.08 |  | 0.71 | 0.150 | 0.44 | 1.13 |
|  | 75-79 (Ref.) | 1.00 |  |  |  |  | 1.00 |  |  |  |  | 1.00 |  |  |  |
|  |  |  |  |  |  |  |  |  |  |  |  |  |  |  |  |
| Dementia | Yes (Ref. No) | 1.17 | 0.022 | 1.02 | 1.35 |  | 1.01 | 0.973 | 0.76 | 1.34 |  | 1.23 | 0.438 | 0.73 | 2.08 |
| Parkinson's disease | Yes (Ref. No) | 1.15 | 0.150 | 0.95 | 1.38 |  | 1.09 | 0.642 | 0.75 | 1.58 |  | 1.24 | 0.545 | 0.62 | 2.49 |
| Heart failure | Yes (Ref. No) | 1.27 | <0.001 | 1.17 | 1.39 |  | 1.86 | <0.001 | 1.49 | 2.33 |  | 1.85 | 0.005 | 1.20 | 2.86 |
| Stroke and/or MI | Yes (Ref. No) | 1.08 | 0.136 | 0.97 | 1.20 |  | 1.07 | 0.596 | 0.84 | 1.36 |  | 1.41 | 0.130 | 0.90 | 2.22 |
| COPD | Yes (Ref. No) | 1.37 | <0.001 | 1.24 | 1.51 |  | 1.50 | <0.001 | 1.19 | 1.89 |  | 1.87 | 0.004 | 1.21 | 2.87 |
| Alcohol abuse | Yes (Ref. No) | 1.46 | <0.001 | 1.21 | 1.76 |  | 1.32 | 0.180 | 0.88 | 1.97 |  | 0.82 | 0.691 | 0.31 | 2.17 |
| Nicotine abuse | Yes (Ref. No) | 1.25 | 0.021 | 1.03 | 1.50 |  | 1.24 | 0.297 | 0.83 | 1.86 |  | 1.03 | 0.952 | 0.42 | 2.49 |
| Rheumatism | Yes (Ref. No) | 1.12 | 0.118 | 0.97 | 1.29 |  | 1.14 | 0.485 | 0.79 | 1.66 |  | 0.99 | 0.973 | 0.48 | 2.05 |
| Diabetes mellitus | Yes (Ref. No) | 1.17 | <0.001 | 1.08 | 1.27 |  | 1.26 | 0.035 | 1.02 | 1.56 |  | 1.39 | 0.125 | 0.91 | 2.10 |
|  |  |  |  |  |  |  |  |  |  |  |  |  |  |  |  |
| Care need level | 0 (Ref.) | 1.00 |  |  |  |  | 1.00 |  |  |  |  |  |  |  |  |
|  | 1 | 1.62 | <0.001 | 1.41 | 1.87 |  | 2.06 | <0.001 | 1.52 | 2.80 |  | 1.83 | 0.029 | 1.06 | 3.15 |
|  | 2 | 2.06 | <0.001 | 1.73 | 2.45 |  | 2.52 | <0.001 | 1.80 | 3.52 |  | 1.28 | 0.458 | 0.67 | 2.47 |
|  | 3 | 3.31 | <0.001 | 2.47 | 4.45 |  | 3.72 | <0.001 | 2.25 | 6.17 |  | 1.65 | 0.348 | 0.58 | 4.72 |
|  |  |  |  |  |  |  |  |  |  |  |  |  |  |  |  |
| Nursing home | Yes (Ref. No) | 1.08 | 0.393 | 0.91 | 1.28 |  | 1.04 | 0.798 | 0.76 | 1.42 |  | 0.61 | 0.144 | 0.31 | 1.18 |
|  |  |  |  |  |  |  |  |  |  |  |  |  |  |  |  |
| Discharge diagnosis | S72.0 (Ref.) | 1.00 |  |  |  |  | 1.00 |  |  |  |  | 1.00 |  |  |  |
|  | S72.1 | 1.14 | 0.030 | 1.01 | 1.29 |  | 1.10 | 0.454 | 0.86 | 1.39 |  | 1.22 | 0.407 | 0.77 | 1.94 |
|  | M16 | 0.47 | <0.001 | 0.42 | 0.53 |  | 0.16 | <0.001 | 0.11 | 0.22 |  | 0.16 | <0.001 | 0.08 | 0.31 |
|  |  |  |  |  |  |  |  |  |  |  |  |  |  |  |  |
| Date of week, surgery | Sunday | 0.88 | 0.217 | 0.71 | 1.08 |  | 0.71 | 0.144 | 0.44 | 1.13 |  | 0.75 | 0.481 | 0.33 | 1.69 |
|  | Monday (Ref.) | 1.00 |  |  |  |  | 1.00 |  |  |  |  | 1.00 |  |  |  |
|  | Tuesday | 1.01 | 0.834 | 0.89 | 1.16 |  | 0.88 | 0.506 | 0.60 | 1.28 |  | 0.65 | 0.251 | 0.31 | 1.36 |
|  | Wednesday | 0.98 | 0.816 | 0.86 | 1.13 |  | 0.98 | 0.930 | 0.69 | 1.41 |  | 0.80 | 0.532 | 0.41 | 1.59 |
|  | Thursday | 0.91 | 0.200 | 0.80 | 1.05 |  | 0.89 | 0.551 | 0.60 | 1.31 |  | 0.83 | 0.613 | 0.41 | 1.69 |
|  | Friday | 1.07 | 0.343 | 0.93 | 1.23 |  | 1.05 | 0.809 | 0.73 | 1.51 |  | 0.74 | 0.407 | 0.37 | 1.50 |
|  | Saturday | 0.83 | 0.086 | 0.67 | 1.03 |  | 0.78 | 0.297 | 0.49 | 1.24 |  | 0.77 | 0.545 | 0.33 | 1.78 |
| Number of patients |  | 6098 |  |  |  |  | 6098 |  |  |  |  | 6098 |  |  |  |
| Number of deaths |  | 2470 |  |  |  |  | 361 |  |  |  |  | 97 |  |  |  |

HR: Hazard ratio, UCI: upper confidence interval, LCI: lower confidence interval, Ref.: Reference group, MI: myocardial infection, COPD: chronic obstructive pulmonary disease, S72.0: femur neck fracture, S72.1: pertrochanteric fracture, M16: coxarthrosis
