# Supplementary material for: Comparing mortality in the elderly after proximal femur fractures and coxarthrosis: the effect of individual health characteristics and day of surgery
Source: Eur J Trauma Emerg Surg. 2025 May 20;51(1):213. doi: 10.1007/s00068-025-02882-y (PMC12092511; doi:10.1007/s00068-025-02882-y)
Supplement: Supplementary file 3 — Supplementary Material 3 [file 68_2025_2882_MOESM3_ESM.docx]

Table S3: Results of Cox regression models for patients aged 80 years and older. Risk of mortality in terms of hazard ratios (HR) 5 years, 1 year and 30 days after hip surgery with 95 % confidence interval. Source: AOK data 2004-2019.

|  |  | **5-year mortality** | | | |  | **1-year mortality** | | | |  | **30-day mortality** | | | |
| --- | --- | --- | --- | --- | --- | --- | --- | --- | --- | --- | --- | --- | --- | --- | --- |
| **Variable** |  | **HR** | **p** | **LCI** | **UCI** |  | **HR** | **p** | **LCI** | **UCI** |  | **HR** | **p** | **LCI** | **UCI** |
| Sex | Women (Ref. Men) | 0.64 | <0.001 | 0.58 | 0.69 |  | 0.55 | <0.001 | 0.48 | 0.63 |  | 0.52 | <0.001 | 0.42 | 0.66 |
|  |  |  |  |  |  |  |  |  |  |  |  |  |  |  |  |
| Age group | 80-84 (Ref.) | 1.00 |  |  |  |  | 1.00 |  |  |  |  | 1.00 |  |  |  |
|  | 85-89 | 1.28 | <0.001 | 1.18 | 1.38 |  | 1.28 | 0.001 | 1.11 | 1.49 |  | 1.49 | 0.004 | 1.14 | 1.96 |
|  | 90+ | 1.70 | <0.001 | 1.55 | 1.85 |  | 1.90 | <0.001 | 1.64 | 2.20 |  | 2.49 | <0.001 | 1.92 | 3.23 |
|  |  |  |  |  |  |  |  |  |  |  |  |  |  |  |  |
| Dementia | Yes (Ref. No) | 1.22 | <0.001 | 1.13 | 1.32 |  | 1.20 | 0.005 | 1.06 | 1.37 |  | 1.15 | 0.195 | 0.93 | 1.43 |
| Parkinson's disease | Yes (Ref. No) | 0.97 | 0.627 | 0.86 | 1.10 |  | 0.87 | 0.192 | 0.71 | 1.07 |  | 1.07 | 0.715 | 0.75 | 1.51 |
| Heart failure | Yes (Ref. No) | 1.18 | <0.001 | 1.10 | 1.26 |  | 1.43 | <0.001 | 1.26 | 1.62 |  | 1.77 | <0.001 | 1.40 | 2.23 |
| Stroke and/or MI | Yes (Ref. No) | 1.21 | <0.001 | 1.12 | 1.30 |  | 1.17 | 0.010 | 1.04 | 1.33 |  | 1.23 | 0.049 | 1.00 | 1.52 |
| COPD | Yes (Ref. No) | 1.03 | 0.447 | 0.95 | 1.13 |  | 1.20 | 0.010 | 1.04 | 1.38 |  | 1.15 | 0.238 | 0.91 | 1.46 |
| Alcohol abuse | Yes (Ref. No) | 0.69 | 0.108 | 0.44 | 1.09 |  | 0.60 | 0.257 | 0.24 | 1.46 |  | 0.90 | 0.885 | 0.22 | 3.71 |
| Nicotine abuse | Yes (Ref. No) | 1.38 | 0.036 | 1.02 | 1.88 |  | 1.19 | 0.502 | 0.71 | 1.99 |  | 1.42 | 0.372 | 0.66 | 3.10 |
| Rheumatism | Yes (Ref. No) | 0.89 | 0.062 | 0.79 | 1.01 |  | 0.88 | 0.264 | 0.71 | 1.10 |  | 0.76 | 0.187 | 0.51 | 1.14 |
| Diabetes mellitus | Yes (Ref. No) | 1.03 | 0.466 | 0.96 | 1.10 |  | 1.03 | 0.585 | 0.92 | 1.16 |  | 1.11 | 0.280 | 0.92 | 1.36 |
|  |  |  |  |  |  |  |  |  |  |  |  |  |  |  |  |
| Care need level | 0 (Ref.) | 1.00 |  |  |  |  | 1.00 |  |  |  |  | 1.00 |  |  |  |
|  | 1 | 1.38 | <0.001 | 1.26 | 1.52 |  | 1.60 | <0.001 | 1.33 | 1.91 |  | 1.67 | 0.001 | 1.23 | 2.27 |
|  | 2 | 1.73 | <0.001 | 1.55 | 1.92 |  | 2.00 | <0.001 | 1.66 | 2.40 |  | 1.74 | 0.001 | 1.27 | 2.38 |
|  | 3 | 2.24 | <0.001 | 1.92 | 2.61 |  | 2.59 | <0.001 | 2.05 | 3.26 |  | 1.24 | 0.354 | 0.79 | 1.95 |
|  |  |  |  |  |  |  |  |  |  |  |  |  |  |  |  |
| Nursing home | Yes (Ref. No) | 1.03 | 0.514 | 0.95 | 1.12 |  | 0.91 | 0.159 | 0.80 | 1.04 |  | 0.83 | 0.114 | 0.67 | 1.04 |
|  |  |  |  |  |  |  |  |  |  |  |  |  |  |  |  |
| Discharge diagnosis | S72.0 (Ref.) | 1.00 |  |  |  |  | 1.00 |  |  |  |  | 1.00 |  |  |  |
|  | S72.1 | 1.00 | 0.971 | 0.93 | 1.08 |  | 1.05 | 0.405 | 0.94 | 1.18 |  | 1.00 | 0.983 | 0.82 | 1.22 |
|  | M16 | 0.49 | <0.001 | 0.44 | 0.55 |  | 0.14 | <0.001 | 0.09 | 0.22 |  | 0.12 | <0.001 | 0.05 | 0.28 |
|  |  |  |  |  |  |  |  |  |  |  |  |  |  |  |  |
| Date of week, surgery | Sunday | 0.88 | 0.075 | 0.77 | 1.01 |  | 0.88 | 0.252 | 0.70 | 1.10 |  | 0.83 | 0.364 | 0.56 | 1.24 |
|  | Monday (Ref.) | 1.00 |  |  |  |  | 1.00 |  |  |  |  | 1.00 |  |  |  |
|  | Tuesday | 1.03 | 0.651 | 0.91 | 1.16 |  | 1.06 | 0.587 | 0.86 | 1.30 |  | 1.18 | 0.358 | 0.83 | 1.66 |
|  | Wednesday | 0.95 | 0.421 | 0.84 | 1.07 |  | 0.96 | 0.682 | 0.78 | 1.18 |  | 1.17 | 0.388 | 0.82 | 1.65 |
|  | Thursday | 0.96 | 0.539 | 0.85 | 1.09 |  | 0.92 | 0.435 | 0.74 | 1.14 |  | 0.98 | 0.929 | 0.68 | 1.42 |
|  | Friday | 0.97 | 0.601 | 0.86 | 1.09 |  | 0.99 | 0.938 | 0.81 | 1.21 |  | 0.92 | 0.668 | 0.64 | 1.32 |
|  | Saturday | 0.92 | 0.195 | 0.80 | 1.05 |  | 0.91 | 0.414 | 0.73 | 1.14 |  | 0.93 | 0.714 | 0.63 | 1.37 |
| Number of patients |  | 4212 |  |  |  |  | 4212 |  |  |  |  | 4212 |  |  |  |
| Number of deaths |  | 3660 |  |  |  |  | 1249 |  |  |  |  | 416 |  |  |  |

HR: Hazard ratio, UCI: upper confidence interval, LCI: lower confidence interval, Ref.: Reference group, MI: myocardial infection, COPD: chronic obstructive pulmonary disease, S72.0: femur neck fracture, S72.1: pertrochanteric fracture, M16: coxarthrosis
