# Supplementary material for: Comparing mortality in the elderly after proximal femur fractures and coxarthrosis: the effect of individual health characteristics and day of surgery
Source: Eur J Trauma Emerg Surg. 2025 May 20;51(1):213. doi: 10.1007/s00068-025-02882-y (PMC12092511; doi:10.1007/s00068-025-02882-y)
Supplement: Supplementary file 4 — Supplementary Material 4 [file 68_2025_2882_MOESM4_ESM.docx]

Table S4: Results of Cox regression models for patients with fracture of femur neck or pertrochanteric fracture. Risk of mortality in terms of hazard ratios (HR) 5 years, 1 year and 30 days after hip surgery with 95 % confidence interval. Source: AOK data 2004-2019.

|  |  | **5-year mortality** | | | |  | **1-year mortality** | | | |  | **30-day mortality** | | | |
| --- | --- | --- | --- | --- | --- | --- | --- | --- | --- | --- | --- | --- | --- | --- | --- |
| **Variable** |  | **HR** | **p** | **LCI** | **UCI** |  | **HR** | **p** | **LCI** | **UCI** |  | **HR** | **p** | **LCI** | **UCI** |
| Sex | Women (Ref. Men) | 0.63 | <0.001 | 0.58 | 0.68 |  | 0.56 | <0.001 | 0.50 | 0.63 |  | 0.53 | <0.001 | 0.43 | 0.65 |
|  |  |  |  |  |  |  |  |  |  |  |  |  |  |  |  |
| Age group | 50-54 | 0.43 | 0.002 | 0.25 | 0.73 |  | 0.19 | 0.096 | 0.03 | 1.35 |  | 0.67 | 0.698 | 0.09 | 5.07 |
|  | 55-59 | 0.44 | <0.001 | 0.30 | 0.64 |  | 0.57 | 0.158 | 0.27 | 1.24 |  | 0.28 | 0.216 | 0.04 | 2.09 |
|  | 60-64 | 0.51 | <0.001 | 0.40 | 0.65 |  | 0.49 | 0.011 | 0.28 | 0.85 |  | 0.34 | 0.074 | 0.10 | 1.11 |
|  | 65-69 | 0.56 | <0.001 | 0.46 | 0.68 |  | 0.72 | 0.087 | 0.49 | 1.05 |  | 0.27 | 0.014 | 0.10 | 0.77 |
|  | 70-74 | 0.73 | <0.001 | 0.64 | 0.84 |  | 0.78 | 0.084 | 0.59 | 1.03 |  | 0.61 | 0.070 | 0.35 | 1.04 |
|  | 75-79 (Ref.) | 1.00 |  |  |  |  | 1.00 |  |  |  |  |  |  |  |  |
|  | 80-84 | 1.20 | <0.001 | 1.09 | 1.33 |  | 1.20 | 0.064 | 0.99 | 1.45 |  | 1.01 | 0.973 | 0.71 | 1.42 |
|  | 85-89 | 1.52 | <0.001 | 1.37 | 1.68 |  | 1.53 | <0.001 | 1.27 | 1.84 |  | 1.51 | 0.014 | 1.08 | 2.09 |
|  | 90+ | 2.00 | <0.001 | 1.80 | 2.22 |  | 2.25 | <0.001 | 1.87 | 2.71 |  | 2.52 | <0.001 | 1.83 | 3.47 |
|  |  |  |  |  |  |  |  |  |  |  |  |  |  |  |  |
| Dementia | Yes (Ref. No) | 1.20 | <0.001 | 1.11 | 1.29 |  | 1.16 | 0.015 | 1.03 | 1.30 |  | 1.14 | 0.207 | 0.93 | 1.39 |
| Parkinson's disease | Yes (Ref. No) | 0.99 | 0.792 | 0.88 | 1.10 |  | 0.92 | 0.402 | 0.77 | 1.11 |  | 1.11 | 0.498 | 0.82 | 1.52 |
| Heart failure | Yes (Ref. No) | 1.20 | <0.001 | 1.12 | 1.28 |  | 1.50 | <0.001 | 1.34 | 1.68 |  | 1.79 | <0.001 | 1.45 | 2.21 |
| Stroke and/or MI | Yes (Ref. No) | 1.16 | <0.001 | 1.08 | 1.24 |  | 1.14 | 0.019 | 1.02 | 1.28 |  | 1.24 | 0.028 | 1.02 | 1.50 |
| COPD | Yes (Ref. No) | 1.15 | <0.001 | 1.07 | 1.24 |  | 1.26 | <0.001 | 1.12 | 1.43 |  | 1.25 | 0.036 | 1.02 | 1.55 |
| Alcohol abuse | Yes (Ref. No) | 1.09 | 0.360 | 0.90 | 1.32 |  | 1.03 | 0.881 | 0.71 | 1.49 |  | 0.84 | 0.679 | 0.37 | 1.90 |
| Nicotine abuse | Yes (Ref. No) | 1.27 | 0.015 | 1.05 | 1.53 |  | 1.26 | 0.156 | 0.91 | 1.75 |  | 1.31 | 0.362 | 0.73 | 2.37 |
| Rheumatism | Yes (Ref. No) | 0.96 | 0.488 | 0.86 | 1.08 |  | 0.95 | 0.590 | 0.78 | 1.15 |  | 0.79 | 0.210 | 0.55 | 1.14 |
| Diabetes mellitus | Yes (Ref. No) | 1.06 | 0.079 | 0.99 | 1.12 |  | 1.06 | 0.291 | 0.95 | 1.17 |  | 1.17 | 0.080 | 0.98 | 1.41 |
|  |  |  |  |  |  |  |  |  |  |  |  |  |  |  |  |
| Care need level | 0 (Ref.) | 1.00 |  |  |  |  | 1.00 |  |  |  |  | 1.00 |  |  |  |
|  | 1 | 1.44 | <0.001 | 1.32 | 1.56 |  | 1.69 | <0.001 | 1.45 | 1.99 |  | 1.72 | <0.001 | 1.32 | 2.26 |
|  | 2 | 1.77 | <0.001 | 1.61 | 1.94 |  | 2.12 | <0.001 | 1.80 | 2.50 |  | 1.71 | <0.001 | 1.29 | 2.26 |
|  | 3 | 2.31 | <0.001 | 2.01 | 2.65 |  | 2.77 | <0.001 | 2.25 | 3.42 |  | 1.30 | 0.213 | 0.86 | 1.97 |
|  |  |  |  |  |  |  |  |  |  |  |  |  |  |  |  |
| Nursing home | Yes (Ref. No) | 1.04 | 0.364 | 0.96 | 1.12 |  | 0.93 | 0.250 | 0.83 | 1.05 |  | 0.81 | 0.058 | 0.66 | 1.01 |
|  |  |  |  |  |  |  |  |  |  |  |  |  |  |  |  |
| Discharge diagnosis | S72.0 (Ref.) | 1.00 |  |  |  |  | 1.00 |  |  |  |  | 1.00 |  |  |  |
|  | S72.1 | 1.03 | 0.304 | 0.97 | 1.10 |  | 1.06 | 0.283 | 0.95 | 1.17 |  | 1.04 | 0.659 | 0.87 | 1.25 |
|  |  |  |  |  |  |  |  |  |  |  |  |  |  |  |  |
|  |  |  |  |  |  |  |  |  |  |  |  |  |  |  |  |
| Date of week, surgery | Sunday | 0.84 | 0.003 | 0.74 | 0.94 |  | 0.82 | 0.052 | 0.67 | 1.00 |  | 0.84 | 0.346 | 0.59 | 1.21 |
|  | Monday (Ref.) | 1.00 |  |  |  |  | 1.00 |  |  |  |  | 1.00 |  |  |  |
|  | Tuesday | 0.96 | 0.504 | 0.86 | 1.08 |  | 0.97 | 0.754 | 0.81 | 1.17 |  | 1.05 | 0.770 | 0.76 | 1.45 |
|  | Wednesday | 0.91 | 0.079 | 0.81 | 1.01 |  | 0.94 | 0.517 | 0.78 | 1.13 |  | 1.12 | 0.483 | 0.81 | 1.55 |
|  | Thursday | 0.85 | 0.005 | 0.76 | 0.95 |  | 0.87 | 0.142 | 0.71 | 1.05 |  | 0.99 | 0.975 | 0.71 | 1.39 |
|  | Friday | 0.94 | 0.243 | 0.84 | 1.04 |  | 0.98 | 0.815 | 0.82 | 1.17 |  | 0.93 | 0.655 | 0.67 | 1.29 |
|  | Saturday | 0.85 | 0.008 | 0.76 | 0.96 |  | 0.86 | 0.149 | 0.71 | 1.05 |  | 0.92 | 0.650 | 0.65 | 1.31 |
| Number of patients |  | 5094 |  |  |  |  | 5094 |  |  |  |  | 5094 |  |  |  |
| Number of deaths |  | 4328 |  |  |  |  | 1517 |  |  |  |  | 490 |  |  |  |

HR: Hazard ratio, UCI: upper confidence interval, LCI: lower confidence interval, Ref.: Reference group, MI: myocardial infection, COPD: chronic obstructive pulmonary disease, S72.0: femur neck fracture, S72.1: pertrochanteric fracture, M16: coxarthrosis
